# Cystic Fluorodeoxyglucose-Avid Thymic Hyperplasia with Lymphoepithelial Sialadenitis-like Features

**DOI:** 10.1093/icvts/ivaf297

**Published:** 2025-12-08

**Authors:** Mahiro Ishizumi, Yoshito Yamada, Shinsuke Shibuya, Akihiro Aoyama

**Affiliations:** Department of Thoracic Surgery, Kyoto Katsura Hospital, Kyoto, Japan; Department of Thoracic Surgery, Kyoto Katsura Hospital, Kyoto, Japan; Department of Diagnostic Pathology, Kyoto Katsura Hospital, Kyoto, Japan; Department of Thoracic Surgery, Kyoto Katsura Hospital, Kyoto, Japan

**Keywords:** anterior mediastinum, autoimmune disease, cystic lesions, fluorodeoxyglucose uptake, thymic MALT lymphoma, thymic hyperplasia with lymphoepithelial sialadenitis-like features

## Abstract

Thymic hyperplasia with lymphoepithelial sialadenitis-like features (LESA-like TH) is a rare benign thymic lesion sometimes associated with autoimmune diseases or mucosa-associated lymphoid tissue lymphoma. A 47-year-old man was incidentally found to have 2 anterior mediastinal masses. Contrast-enhanced computed tomography (CT) showed irregularly walled cysts, and positron emission tomography-CT demonstrated partial fluorodeoxyglucose (FDG) uptake. A total thymectomy was performed, and histopathology confirmed LESA-like TH. This is the first report to document both imaging-visible multiplicity and FDG avidity in LESA-like TH, which broadens the recognized imaging spectrum of this rare thymic lesion.

## INTRODUCTION

Thymic hyperplasia with lymphoepithelial sialadenitis-like features (LESA-like TH) was first characterized in 2012,^1^ as a rare benign thymic variant. Fewer than 50 cases have been reported. It is occasionally linked to autoimmune disease and thymic mucosa-associated lymphoid tissue (MALT) lymphoma.^2^ Histologically, it resembles lymphoepithelial sialadenitis of the salivary glands.^3^ However, its radiologic features remain poorly defined. We report a case of cystic LESA-like TH showing fluorodeoxyglucose (FDG) avidity and discuss its radiologic and histologic characteristics.

## CASE PRESENTATION

We adhered to the ethical standards of Kyoto Katsura Hospital and obtained Institutional Review Board approval (No. 2025–39; August 25, 2025). Written informed consent for publication was obtained from the patient.

**Figure 1.**
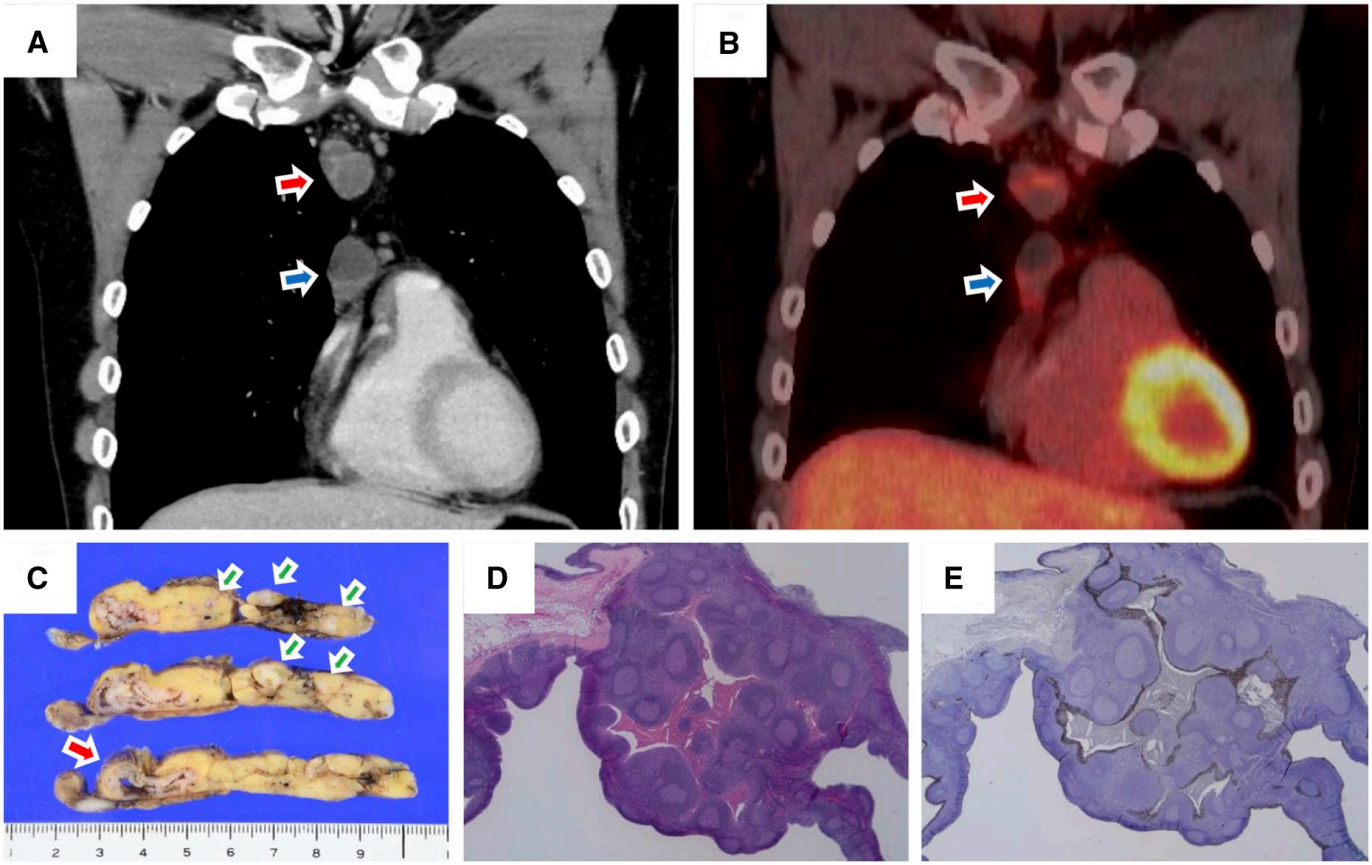
Radiologic and Pathologic Findings. (A) Contrast-enhanced CT showing cysts with mildly enhancing walls. (B) PET-CT showing two lesions (SUV_max_ 3.3 and 3.6; red and blue arrows). (C) Cut surface of thymus with a cystic cavity (red arrow) and scattered nodules (green arrow). (D) H&E showing cyst lined by epithelium with lymphoid follicular hyperplasia and cholesterol clefts. (E) Cytokeratin 19 highlights cyst epithelium

A 47-year-old man undergoing computed tomography (CT) for acute gastroenteritis was incidentally found to have two anterior mediastinal masses (29 × 18 and 37 × 19 mm). Contrast-enhanced CT revealed cysts with partial wall enhancement, and positron emission tomography (PET)-CT demonstrated mild FDG uptake (**Figure 1A and B**). Considering possible malignancy, a total thymectomy was performed.

Grossly, the thymus showed fatty replacement with two cystic lesions and numerous tiny grayish-white nodules (**Figure 1C**). Histologically, the cyst walls displayed lymphoid follicular hyperplasia, lymphoepithelial lesions, and eosinophilic material with cholesterol clefts (**Figure 1D**). The scattered small white nodules represented multifocal microscopic cystic lesions with similar histologic findings. Immunohistochemically, the cystic epithelium was positive for AE1/AE3 and cytokeratin 19 (**Figure 1E**). The lymphoid follicles exhibited a reactive pattern confirmed by CD3, CD20, CD10, Bcl-2, CD21, and Ki-67 expression. Terminal deoxynucleotidyl transferase-positive immature T cells were rare. Based on overall pathological features, the final diagnosis was LESA-like TH. The reactive lymphoid follicles, along with the absence of specific findings on flow cytometry, karyotyping, and genomic Southern blot analysis, excluded lymphoma. Autoimmune screening showed no abnormalities, and the patient has remained recurrence-free one year postoperatively.

## DISCUSSION

We report a case of LESA-like TH, a rare benign thymic lesion. Although several cases have been reported (**Table 1**), it remains uncommon. Radiologically, LESA-like TH usually appears as a well-circumscribed mediastinal mass that may be solid, cystic, or mixed, sometimes showing internal septa and mild enhancement. Histologically, it is characterized by multifocal lymphoepithelial cyst-like changes scattered throughout the thymus, reactive lymphoid follicles with germinal centers, abundant thymic epithelium with Hassall corpuscles, and polyclonal lymphocytes or plasma cells without cytologic atypia or invasive growth. Differentiation from thymic epithelial tumors or lymphoma is crucial, as they can share overlapping radiologic and histologic features, whereas LESA-like TH shows no atypia or invasion. In our case, mildly FDG-avid cystic lesions were observed, which initially suggested malignancy. However, the uptake likely reflected inflammatory and hyperplastic activity rather than cancer. Therefore, PET-CT findings should always be interpreted with caution.

**Table 1.** Summary of Reported Cases of LESA-like TH

References	No. of cases	Sex (M/F)	Median age (range)	Median size (range), mm	Cases of Autoimmune disorders	Lymphoma association	Radiological findings
Weissferdt et al^1^	4	2/2	45.5 (37-53)	162.5 (115-210)	0	0	**CT**: anterior mediastinal mass
Porubsky et al^3^	36	21/15	51.5 (32-80)	70 (10-145)	12 ^a^	5 ^c^	**CT/MRI**: mediastinal mass
Arndt et al^5^	1	1/0	43	40	0	0	**CT**: inhomogeneous mass with ill-defined margins and peripheral calcifications
Tanaka et al^4^	1	1/0	55	20	1 ^b^	0	**CT**: mass with mild enhancement **MRI**: cystic change on T2
Xu et al^2^	1	0/1	52	60	0	0	**CT**: cystic-solid mass with septa and mild–moderate enhancement
Current case	1	1/0	47	37, 29	0	0	**CT**: two cystic lesions with wall enhancement **PET**: mild FDG uptake

All references summarized here are indexed in PubMed.

a 4 systemic lupus erythematosus, 3 rheumatoid arthritis, 2 myasthenia gravis, 1 Sjögren disease, 1 scleroderma, 1 pure red cell aplasia, 1 Graves’ disease, 1 anti-IgLON5 syndrome.

b The IgG4-related disorder.

c 4 Thymic MALT lymphomas, 1 diffuse large B-cell lymphoma.

Data on the long-term outcomes of LESA-like TH are limited, and no recurrences have been reported, but its behaviour remains unclear. Therefore, further accumulation of cases is necessary. In the largest cohort to date, 33% of patients had concurrent autoimmune diseases, and 14% were associated with thymic MALT lymphoma,^3^ supporting an inflammatory and immune-mediated background that warrants attention during follow-up. We suggest annual CT surveillance for several years after surgery, followed by symptom-driven evaluation thereafter. Baseline screening and patient education are recommended to facilitate early recognition and appropriate assessment if autoimmune symptoms arise.

## CONCLUSION

We encountered a rare case of LESA-like TH and discussed its imaging and histologic findings in the context of previously reported cases.

## Data Availability

All data are in the article.
